# *O*-Mucin-degrading carbohydrate-active enzymes and their possible implication in inflammatory bowel diseases

**DOI:** 10.1042/EBC20220153

**Published:** 2023-04-18

**Authors:** Aurore Labourel, Jean-Luc Parrou, Céline Deraison, Muriel Mercier-Bonin, Sophie Lajus, Gabrielle Potocki-Veronese

**Affiliations:** 1 TBI, Université de Toulouse, CNRS, INRAE, INSA, Toulouse, France; 2 IRSD, Université de Toulouse, INSERM, INRAE, ENVT, Univ Toulouse III - Paul Sabatier (UPS), Toulouse, France; 3 Toxalim, Research Center in Food Toxicology, INRAE, ENVT, INP-Purpan, UPS, Université de Toulouse, 31000, Toulouse, France

**Keywords:** Cazymes, IBD, metagenomics, microfluidics, O-mucins

## Abstract

Inflammatory bowel diseases (IBD) are modern diseases, with incidence rising around the world. They are associated with perturbation of the intestinal microbiota, and with alteration and crossing of the mucus barrier by the commensal bacteria that feed on it. In the process of mucus catabolism and invasion by gut bacteria, carbohydrate-active enzymes (CAZymes) play a critical role since mucus is mainly made up by *O*- and *N*-glycans. Moreover, the occurrence of IBD seems to be associated with low-fiber diets. Conversely, supplementation with oligosaccharides, such as human milk oligosaccharides (HMOs), which are structurally similar to intestinal mucins and could thus compete with them towards bacterial mucus-degrading CAZymes, has been suggested to prevent inflammation. In this mini-review, we will establish the current state of knowledge regarding the identification and characterization of mucus-degrading enzymes from both cultured and uncultured species of gut commensals and enteropathogens, with a particular focus on the present technological opportunities available to further the discovery of mucus-degrading CAZymes within the entire gut microbiome, by coupling microfluidics with metagenomics and culturomics. Finally, we will discuss the challenges to overcome to better assess how CAZymes targeting specific functional oligosaccharides could be involved in the modulation of the mucus-driven cross-talk between gut bacteria and their host in the context of IBD.

## Introduction

Intestinal mucus is notably formed by glycoproteins, the *O*- and *N*-linked glycans which make up to 80% of their total mass. These structurally complex glycans influence the cohesiveness of the mucus network. Their structure and defects in their biosynthesis [1] may be differently modulated by environmental factors [2–4], in particular in inflammatory bowel diseases (IBD), such as Crohn’s disease (CD) and ulcerative colitis (UC). These diseases, which are more prevalent in the modern world, are associated with the Western diet with low gut microbiota-accessible carbohydrates, which is linked to a lack of microbial diversity and altered production of short-chain fatty acids [5]. IBD have become increasingly prevalent in Europe, North America and Australia since the 1950s, with their incidence rising beyond the western world over the past 30 years [6]. They are characterized by an imbalance in the composition of the intestinal microbiota [7–9], the so-called ‘dysbiosis’, and by an uncontrolled inflammatory response to luminal content [10]. In addition, the intestinal barrier function is severely impaired: significant changes in mucus secretion and mucin *O*-glycosylation occur, the inner mucus barrier is thinner and looser, and the space closest to the epithelium is invaded by commensal bacteria and/or enteropathogens [11–14]. Indeed, together with the dietary glycans (HMOs for infants, and then fibers of plant origin) and, to a minor extent, with microbial exopolysaccharides, the mucus glycans are the main carbon sources for gut bacteria. To deal with the huge structural diversity of glycans they feed on, and to break them down into metabolizable monosaccharides, intestinal bacteria produce a large panel of carbohydrate-active enzymes (CAZymes) with various specificities [15], and a battery of proteins to sense, bind and transport glycans into bacterial cells for their complete breakdown. These metabolic machineries are often encoded by multigenic clusters, defined as polysaccharide utilization loci (PUL) in the Gram-negative Bacteroidota (formerly Bacteroidetes [16]), one of the dominant phyla in the human gut. Bacteroidota include bacteria that are able to feed on mucins, such as the commensal species *Bacteroides thetaiotamicron* and *Bacteroides fragilis*, the abundance of which seems to be correlated to CD and UC [17,18]. CAZymes targeting mucins are also found in other Gram-negative mucin degraders, such as, for example, *Akkermansia muciniphila* [19], which is negatively associated with IBD [8], and in Gram-positive ones, such as *Ruminococcus gnavus*, which is, on the contrary, positively associated with these diseases, and CD in particular [20]. Given that mucin degraders can be both positively and negatively correlated to IBD, it is very difficult to determine whether mucin breakdown by gut bacterial CAZymes is involved in intestinal epithelium inflammation. CAZymes produced by mucin-degrading bacteria have been reviewed several times over the last decade [21–25]. Nevertheless, some recent functional metagenomics studies have highlighted the potential of CAZymes produced by uncultured gut bacteria, and appearing as IBD biomarkers, to degrade human *O*-glycans present in mucus [26,27] and in human milk oligosaccharides (HMOs). These oligosaccharides could indeed compete with the *O*-glycans of mucins lining the intestinal epithelium towards mucus-degrading CAZymes, since they are structurally similar to their extremities. HMOs seem to protect against IBD [28–30], although these data are still controversial [31], as for other functional foods that are metabolized thanks to CAZymes from the intestinal microbiome [32].

In this review, we will update the list of CAZymes that have been shown to target *O*-mucins, since mucins are much more sparsely *N*-glycosylated than *O*-glycosylated [33,34]. Here, we will discuss the advantages and limits of the methods that are currently available to identify them within the entire microbiome. Finally, we will explore the potential role that mucin-degrading CAZymes could play in the interrelationships between the host, diet and the microbiota in the context of gut inflammation.

## Mucin-degrading CAZymes

### Structure of the O-mucins

MUC2, the major colonic mucin is composed of ∼20% protein and ∼80% glycan. The protein sequence rich in Ser/Thr residues are primary targets for *O*-glycosylation with *N*-acetyl-d-galactosamine (GalNAc), creating the foundation upon which long oligosaccharide chains are built. While these side chains are composed of only five different monosaccharides (*N*-acetyl-d-glucosamine (GlcNac), GalNac, d-galactose, l-fucose and *N*-acetylneuraminic acid [also called sialic acid, Neu5Ac]), the order in which they can be assembled is hugely variable. In addition, the GlcNac and galactose residues can be sulfated, adding a supplemental level of complexity. The resulting *O*-glycans extend out from the mucin protein core in a ‘bottlebrush’ configuration and its heterogeneity provides some resistance to microbial degradation [33,35]. It should be emphasized that the mucin *O*-glycans vary enormously along the intestinal tract and between species (Table 1), compelling researchers in the area to take greater care when studying bacteria and mucin from the same niche.

**Table 1:** Main characteristics of substrates

Substrates categories	Representative structures
**Chromogenic /fluorogenic glycoside**	
Origin: synthetic substrates Commercial price: a few hundreds to a few thousand €/g Applications: conventional screening; specificity characterization in microplates; droplet Microfluidics-based screening (fluorogenic substrates)	X-GlcNAc, X-sulfate, X-Neu5Ac, … * p*NP-Fuc, *p*NP-GlcNAc, … Fluorescein-GlcNAc, …
**HMOs and blood antigens**	
Origin: synthetic substrates Commercial price: tens of thousands €/g Applications: specificity characterization (HPAEC-PAD); droplet microfluidics-based screening and culturomics	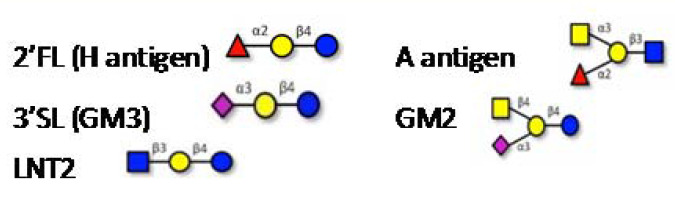
**Pig gastric mucin (PGM)**	
Origin: sampling on animals Commercial price: €1 to €10/g Applications: transcriptomics; characterization of synthetic cocktails of CAZmes	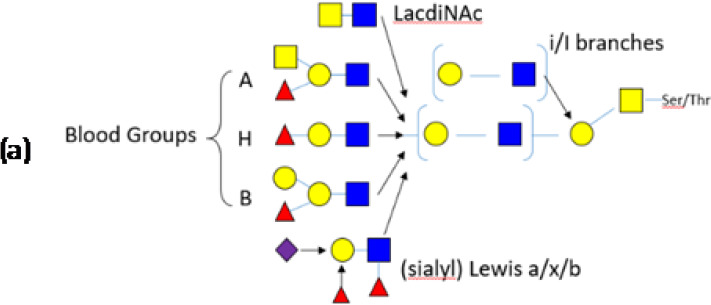
**Human colonic mucin**	
Origin: surgical pieces, cell lines, organoids Not commercial Applications: ELISA assays; characterization of synthetic cocktails of CAZymes (though not exemplified yet)	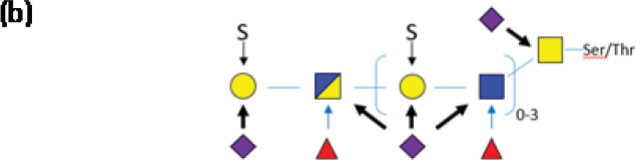

Symbols: GlcNac 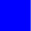, Gal 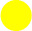, GalNAc 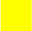, Fucose 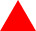, Sialic acid 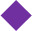, Glc 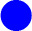, Sulfate **S**; (a) Representative, not exhaustive structures decorating PGM chains (mainly 4–6 units), leading to extreme diversity. (b) Schematic view of the most common, core 3-based branch (adapted from [36]). For the sake of clarity, the nature of the bonds between the units are not depicted.

### Identification and characterization of O-mucin degrading CAZymes

Several comparative genomic analyses have attempted to determine the CAZyme mucin-degrading profiles of the human gut microbiota [37,38], and there have been efforts to couple these data with transcriptomic, proteomic, genetic and biochemical studies in order to understand mucin catabolic pathways. One of the earliest and most studied commensal bacteria is *B. thetaiotaomicron*, which is one of the richest human bacteria in glycan-degrading enzymes [15,39]. In 2008, Martens and his team performed whole-genome transcriptional profiling and genetic tests to define the mechanisms underlying host glycan foraging *in vivo* (mice) and *in vitro* [40]. Mucin *O*-glycans are the principal substrate foraged *in vivo* and 16 PULs were identified with probable specificities for *O*-glycans. Recently, two of these PULs were characterized highlighting the major role of GH16 enzymes [41] and sulfatases [42–44]. Growth assays on mucins combined with transcriptomic analyses were also performed on other Bacteroidota such as *B. fragilis* [45,46] and *B. massiliensis* [45]. Similar studies were performed on the mucin degraders *A. muciniphila* [47–50] (Verrucomicrobia), *R. gnavus* [51] (Firmicutes) and *Bifidobacterium bifidum* [52] (Actinobacteria). This list is destined to get longer as 23 representative gut microbes were shown to grow on porcine intestinal mucin as their sole carbon source [38].

The *O*-mucin-degrading CAZymes identified by genomic and transcriptomics analysis are usually biochemically characterized using porcine gastric and intestinal mucins, bovine submaxillary mucin, mouse crude mucin as well as HMOs, blood-group oligosaccharides and synthetic oligosaccharides. To date, 18 glycoside hydrolase (GH) families were shown to be involved in the degradation of *O*-mucin glycans (www.cazy.org). GH33 sialidases cleave terminal sialic acid residues while fucose residues are removed by GH29 or GH95. Subsequent degradation of the glycan chains involves lacto-*N*-biosidase (GH136), lacto-*N*-biose phosphorylases (GH112), *N*-acetyl-glucosaminidases (GH84, GH85, GH89, GH20), *N*-acetyl-galactosaminidases (GH31, GH101, GH109, GH129) and galactosidases (GH2, GH35, GH42, GH98). There are also endo-acting *O*-glycanases (GH16), which can cleave large glycan structures. Table 2 lists the characterized *O*-mucin-degrading CAZymes since 2015. It is an update of a previous review [22]. To provide an overview of the complete catabolic pathway of *O*-mucins, the proteases that have been shown to be specific to this substrate are listed in Table 3, and the sulfatases and the sialate *O*-acetylesterase are listed in Table 4.

**Table 2:** The list of biochemically characterized *O*-mucin degrading CAZymes

CAZy family	Name or locus tag	Activity	Organism	Accession numbers
GH2	Amuc_0824	β-galactosidase (preference for galactose-β-1,3-*N*-acetyl-d-galactosamine)	*Akkermansia muciniphila* ATCC BAA-835	ACD04657.1 [53]
	Amuc_1666	β-galactosidase (preference for galactose-β-1,4-*N*-acetyl-d-glucosamine)		ACD05484.1 [53]
GH16	BT2824	*O*-Glycan β-1,4-galactosidase with a requirement for a β1,3-linked sugar at the -2 position	*Bacteroides thetaiotaomicron* VPI-5482	AAO77930.1 [41]
	BF4058		*Bacteroides fragilis* NCTC 9343	CAH09732.1 [41]
	BF4060			CAH09734.1 [41] PDB 6T2S
	BACCAC_02679; CGC64_00695		*Bacteroides caccae* ATCC 43185	ASM64646.1 [41]
	BACCAC_02680; CGC64_00690			ASM64645.1 [41] PDB 6T2Q, 6T2P, 2T2O
	BACCAC_03717; CGC64_17650			ASM67518.1 [41] PDB 6T2R
	Amuc_0724		*Akkermansia muciniphila* ATCC BAA-835	ACD04561.1 [41] PDB 6T2N
	Amuc_875			ACD04708.1 [41]
	Amuc_2108			ACD05917.1 [41]
GH29	*Ri*Fuc29, ROSEINA2194_01891/01890	α-1,4-fucosidase	*Roseburia inulinivorans* DSM 16841	C0FT24/C0FT23 (Uniprot) [54]
	E1_10125; RUGNEv3_10125	α-l-fucosidase (preference for α1,3/4 fucosylated linkages)	*Ruminococcus gnavus* E1	A0A6N3BKT0 [55] PDB 6TR3, 6TR4
	E1_10180; RUGNEv3_10180			- [55]
	ATCC_03833; RUMGNA_03833	α-l-fucosidase (preference for α1,2 linkages)	*Ruminococcus gnavus* ATCC 29149	- [55]
	Afc2; CPF_2130	α-l-fucosidase (preference for α1,3/4 fucosylated linkages)	*Clostridium perfringens* ATCC 13124	ABG83106.1 [56]
	*Am*GH29A, Amuc_0010	α-l-fucosidase (preference for Fucα1,3GlcNAc)	*Akkermansia muciniphila* ATCC BAA‐835	ACD03857.1 [57]
	*Am*GH29B, Amuc_0146			ACD03990.1 [57]
	*Am*GH29C, Amuc_0392	α-l-fucosidase (preference for α1,3/4 fucosylated linkages)		ACD04231.1 [57]
	*Am*GH29D, Amuc_0846			ACD04679.1 [58]
GH31	*Bp*GH31	α-*N*-acetylgalactosaminidase	*Phocaeicola plebeius* (formerly *Bacteroides plebeius*)	WP_007559952.1 [58]
	*Bc*GH31		*Bacteroides caccae*	WP_005682123.1 [58]
GH33	*Rg*NanH	Intramolecular *trans*-sialidase (IT-sialidase) producing 2,7-anhydro-Neu5Ac selectively from α2-3-linked sialic acid substrates	*Ruminococcus gnavus* ATCC 29 149	ETD19277.1 [59]
	NanH, BF1806	Sialidase	*Bacteroides fragilis* NCTC 9343	WP_010992682.1 [60]
	*Am*GH33A, Amuc_0625	α2,3‐ and α2,6‐sialyl linkages	*Akkermansia muciniphila* ATCC BAA‐835	ACD04462.1 [57]
	*Am*GH33B, Amuc_1835			ACD05653.1 [57]
GH35	*Am*0874; Amuc_0771	β-galactosidase (β1,3- and β1,6-linked galactose residues from various substrates, preference for galactose-β-1,3-*N*-acetyl-d-glucosamine)	*Akkermansia muciniphila* ATCC BAA-835	ACD04606.1 [53,61]
	Amuc_1686	β-galactosidase (Galβ1-3GalNAc)		ACD05504.1 [62]
GH95	*Ri*Fuc95, ROSEINA2194_01889/01888/01887	α-1,2-fucosidase	*Roseburia inulinivorans* DSM 16841	C0FT22/C0FT21/C0FT2 (Uniprot) [54]
	ATCC_00842; RUMGNA_00842	α-l-fucosidase (preference for α1,2 linkages)	*Ruminococcus gnavus* ATCC 29149	[55]
	E1_10587; RUGNEv3_10587	α-l-fucosidase (low activity, not possible to assess its substrate specificity)	*Ruminococcus gnavus* E1	[55]
	Afc3; CPF_2129	α-l-fucosidase (preference for α1,2 linkages)	*Clostridium perfringens* ATCC 13124	ABG82552.1 [56]
	*Am*GH95A, Amuc_0186	α-l-fucosidase (preference for α1,2/3 fucosylated linkages)	*Akkermansia muciniphila* ATCC BAA‐835	ACD04030.1 [57]
	*Am*GH95B, Amuc_1120			ACD04946.1 [57]
GH98	*Ri*GH98, ROSEINA2194_02198	Blood-group endo-β-1,4-galactosidase	*Roseburia inulinivorans* DSM 16841	C0FTX7 (Uniprot) [54]
GH109	*Bv*GH109_1	α-*N*-acetylgalactosaminidase	*Bacteroides vulgatus*	WP_005848863.1 [58]
GH112	*Rh*GLnbp112, RHOM_04120	β-1,3-galactosyl-*N*-acetylhexosamine phosphorylase	*Roseburia hominis* A2-183	AEN95946.1 [54]
	*Ri*GLnbp112, ROSEINA2194_01885		*Roseburia inulinivorans* DSM 16841	EEG94248.1 [54]
GH136	*Rh*Lnb136I, RHOM_04110 + *Rh*Lnb136II, RHOM_04115	Lacto-*N*-biosidase	*Roseburia hominis* A2-183	G2T0V0 + G2T0V1 (Uniprot) [54]
	*Ri*Le^a/b^_I_136, ROSEINA2194_01899 + *Ri*Le^a/b^_II_136, ROSEINA2194_01898	Lacto-*N*-biosidase. Releases Lewis a triose or Lewis b tetraose from fucosylated HMOs including lacto-*N*-fucopentaose II, lacto-*N*-difucohexaose I and lacto-*N*-difucohexaose II	*Roseburia inulinivorans* DSM 16841	C0FT31 + C0FT32 (Uniprot) [54]
	*Er*Lnb136, HMPREF0373_02965	Lacto-*N*-biosidase	*Eubacterium ramulus* ATCC 29099	ERK42296.1 [54] PDB 6KQS, 6KQT
New family to be assigned	Amuc_1547	Exclusive specificity towards the sialyl‐T‐antigen but accepts α2,6‐sialyation or β1,6‐substitution of the GalNAc unit	*Akkermansia muciniphila* ATCC BAA‐835	ACD05368.1 [57]

**Table 3:** The list of proteases that have been shown to be involved in *O*-mucin degradation

Protease family	Name or locus tag	Activity	Organism	Accession numbers
Serine protease Pic (Protein involved in Intestinal Colonization) autotransporter	Pic	Active on bovine submaxillary mucin and mouse crude mucin. Similar cleavage pattern to BT4244 but no tolerance for the presence of sialic acid	*Escherichia coli* (EAEC) 042	AF097644.1 [63]
	Pic	Active on bovine submaxillary mucin and mouse crude mucin	*Shigella flexneri* 2457T	Q54151 (Uniprot) [63]
M60-like zinc metalloprotease	BT4244	Cleaves peptides N-terminally to glycosylated Ser or Thr residues and preferred the modified residue to bear atruncated, tumor-associated glycan such as the Tn (*O*-GalNAc) or T (*O*-GalNAc-galactose) antigen. Sensitivity to sialic acid	*Bacteroides thetaiotaomicron* VPI-5482	WP_008764444.1 [64–66] PDB 5KD2, 5KD5, 5KD8
	SslE	Active on bovine submaxillary mucin	*Escherichia coli* IHE3034	WP_001034562.1 [67]
	YghJ	Active on human MUC2 and MUC3	*Escherichia coli* H10407 ETEC	E3PJ90 [68] (Uniprot)
	AM0627	Cleaves between two *O*-glycosylated Ser or Thr residues bearing atruncated, tumor-associated glycan such as the Tn (*O*-GalNAc) or T (*O*-GalNAc-galactose) antigen. Sensitivity to sialic acid	*Akkermansia muciniphila* ATCC BAA-835D-5	B2UPI7 (Uniprot) [42,69,70] PDB 7SCI, 7YX8
	AM0908	Cleaves N-terminally to a glycosylated Ser or Thr with a marked preference for an adjacent Ser or Thr residue. Tolerate sialic acid		B2UQK5 (Uniprot) [66]
	AM1514	Cleaves N-terminally to a glycosylated Ser or Thr with a marked preference for an adjacent Ser or Thr residue. Specific for *O*-GalNAc-galactose and *O*-GalNAc antigen-modified amino acids.		B2ULE8 (Uniprot) [66]
	PA0572, IMPa	Hydrolyze a minimally *O*-glycosylated substrate with only the Tn- or TAg-glycans. Highest affinity when this core modification is extended with a β-1,6-linked GlcNAc (GlcNAcβ1-6GalNAcα1-Thr).	*Pseudomonas aeruginosa* (PAO1)	Q9I5W4 (Uniprot) [65,71] PDB 5KDV, 5KDW, 5KDX
	ZmpB, CPF_1489	Absolute requirement for the α-2,6-Neu5Ac present on the peptide-linked GalNAc.	*Clostridium perfringens* ATCC 13124	A0A0H2YN38 (Uniprot) [65,72] PDB 5KDJ, 5KDN, 5KDS, 5KDU
	ZmpC, CP4_3468	Action on similar protein than ZmpB 1 (75% sequence identity)	*Clostridium perfringens* CP4	F8UNJ8 (Uniprot) [72] PDB 6XSZ, 6XT1
M66-like zinc metalloprotease	StcE	Reduce the inner mucus layer in human colonic mucosal biopsies and the MUC2 glycoprotein levels in mucin-producing LS174T colon carcinoma cells	*Escherichia coli* (EHEC)	O82882 (Uniprot) [73]
Peptidase_M43 domain-containing protein	OgpA, Amuc_1119, OpeRATOR® and GlycOCATCH®	Active on Fetuin and IgA. Exclusively hydrolyzes the peptide bond N-terminal to serine or threonine residues substituted with an *O*-glycan	*Akkermansia muciniphila* ATCC BAA‐835	ACD04945.1 [74] PDB 6Z2D, 6Z2O

**Table 4:** The list of sulfatases and sialate* O*-acetylesterase that have been shown to be involved in *O*-mucin degradation

Sulfatase family	Name or locus tag	Activity	Organism	Accession numbers
Sulfatase S1_4	BT3487	6S-Gal	*Bacteroides thetaiotaomicron* VPI-5482	AAO78593.1 [43]
	BT4683	3S-Gal		AAO79788.1 [43] PDB 7ALL
Sulfatase S1_11	BT1628	6S-GlcNAc	*Bacteroides thetaiotaomicron* VPI-5482	AAO76735.1 [43]
	BT3177			AAO78283.1 [43,44] PDB 7P24
Sulfatase S1_15	BT1624	6S-Gal/GalNAc	*Bacteroides thetaiotaomicron* VPI-5482	AAO76731.1 [43,44] PDB 7OZE
	BT3109			AAO78215.1 [43,44] PDB 7OZC
	BT4631			AAO79736.1 [43,44] PDB 7P26
Sulfatase S1_16	BT3057	4S-Gal/4S-GalNAc	*Bacteroides thetaiotaomicron* VPI-5482	AAO78163.1 [43,44] PDB 7OZ9
	BT3796			AAO78901.1 [43,44] PDB 7OZA
Sulfatase S1_20	BT1622	3S-Gal preferentially binding *N*-acetyl-d-galactosamine (GalNAc) and cleaving 3S-GalNAc	*Bacteroides thetaiotaomicron* VPI-5482	AAO76729.1 [43] PDB 7ANB, 7ANA
	BT1636	3S-Gal required enzyme to access colonic mucin by *Bth*		AAO76743.1 [43] PDB 7AN1, 7OQD
Sulfatase S1_46	BT1918	3S-GlcNAc, using the *N*-acetyl group as an absolute specificity determinant	*Bacteroides thetaiotaomicron* VPI-5482	AAO77025.1 [43,44] PDB 7OZ8
Sialate *O*-acetylesterase	EstA	Active on 9-*O*-acetylated sialoglycans	*Bacteroides fragilis* NCTC 9343	WP_005794991.1 [60]

Overall, about a hundred mucin-degrading CAZymes have been biochemically characterized with substrates of defined structure, allowing the determination of their specificity towards the host and dietary glycans that could compete to feed gut bacteria. This limits the understanding, at the molecular level, of the relationships between the microbiota, its host and diet. In addition, these biochemical data are far from being exhaustive, especially when we consider that uncultured species dominate in the human gut microbiota [75]. Recently, activity-based metagenomics [76] have enabled the capture of the first CAZymes from uncultured gut bacteria (α-fucosidases, β-*N*-acetyl-galactosidases, β-galactosidases, β-*N*-acetyl-glucosidases, β-*N*-acetyl-neuraminidases, α- and β-mannosidases), which have proved to affect the structure of various *O*-, *N*-glycans, HMOs and human colonic mucus [26,27,77]. These studies also provided the first evidence of the over-representation of the genes coding for some of these enzymes in the human gut metagenome of patients suffering from IBD.

It is noteworthy that metagenome assemblies generated from the gut microbiota include a wide range of other organisms, apart from bacteria, such as archaea, eukaryotes and viruses that warrant a more thorough investigation [78]. For instance, gut commensal fungi are likely involved in IBD [79] but to our knowledge, none of their CAZymes has been characterized to date.

## Technological bottlenecks and challenges in the discovery and characterization of mucin-degrading CAZymes

The combination of growth assays on mucins combined with transcriptomics, or proteomics [80] is currently the best strategy to highlight the CAZymes involved in mucin degradation by targeted species. These species tend to be either highly prevalent commensals or species that are associated with intestinal diseases [51].

In addition, this approach can be used to identify the enzymes from bacterial mini-consortia that act synergistically to break down the mucin network, or, on the contrary, that compete to forage on mucin glycans [81]. It is nevertheless restricted to the study of cultured species, and although ‘artificial’ consortia could be studied, they do not represent the diversity and the complexity of the bacterial and enzymatic machineries that are involved in mucus breakdown in the gut microbiome. Although those that exist are extremely interesting, there are currently very few metatranscriptomic and/or metaproteomic studies available [82,83] giving access to the battery of CAZyme-encoding genes expressed during mucin consumption by the entire microbiota, in particular in an IBD context [84].

Technically speaking, performing growth assays and transcriptomics or proteomics studies with biological and technical replicates requires grams of substrate, which is easy when such studies are performed on widely available natural substrates such as plant cell wall polysaccharides. When the focus of the studies is human mucin degradation, the challenge is very different because of the impossibility of sampling human intestinal mucus in large amounts. The solution currently used is to perform growth studies on porcine mucins, which share structural similarities with human mucin core proteins. Unfortunately, however, there are important differences in the relative abundance of core types, neutral, acidic or sulfated glycans [85] (Table 1), which are crucial in terms of enzymatic accessibility to the network of mucins. In brief, the main structural characteristics that differentiate pig gastric mucin (PGM) – one of the mostly used substrates for *in vitro* studies – from human colonic mucin are respectively [36,86–88]: MUC5 dominant versus MUC2 enriched mucin; cores 2 and 1 versus core 3 dominant; low degree of sialylation versus extensive sialylation (mono, di tri) and sulfate groups, leading to acidic human colonic mucus; extensive presence of blood type ABH and Lewis epitopes and i/I branches versus extensive presence of the Sda/Cad antigen (GalNAc(NeuAc-)Gal) and low abundance, mono-fucosylation, including blood group ABH antigens; galactose as the most common terminal residue, as well as presence of a sulfate group, approximately 50% of termini for both in PGM. In addition, using such complex polymeric structures does not allow for identification of the specificity of the expressed CAZymes towards a particular oligosaccharidic motif or linkage type, except using powerful but complex techniques combining high-performance anion-exchange chromatography with pulsed amperometric detection (HPAE-PAD), mass spectrometry and liquid chromatography-mass spectrometry (LC-MS) to analyze the structure of the oligosaccharides that are released during bacterial consumption of mucins or their *in vitro* enzymatic hydrolysis [41,89].

Screening and characterizing the substrate specificity of CAZymes can be easily performed using chromogenic glycosides mimicking the mucin chain extremities, such as *p*NP- or X-sugars. However, there are some mucin-acting CAZymes that do not act on these artificial substrates, leading to false negative results. It is the case, for example, of the AfcA GH95 α-fucosidase from *Bifidobacterium longum* JCM1254, which is inactive on *p*NP-fucose [90]. Other GH95 and GH29 α-fucosidases act more or less efficiently on this substrate, with catalytic efficiencies varying by up to four orders of magnitude between GH29 enzymes [55].

On the contrary, the rate of false positives might be high in screening campaigns using unpurified enzymes and chemically modified substrates, that can be recognized by the native glycosidases produced by the recombinant host. For example, when screening *Escherichia coli* recombinant libraries for β-galactosidase activity, the LacZ GH2 β-galactosidase is highly problematic. *E. coli* also produces other glycosidases, such as the GH3 AWY88947 β-*N*-acetylhexosaminidase, resulting in background activity that has to be systematically taken into account to get information on the ability, or not, of the target enzymes to hydrolyze the substrates under study [27].

This is of particular importance for screening genomic or metagenomic libraries for activities of mucin hydrolysis, using conventional solid-plates or micro-plate screens. Using a battery of chromogenic glycosides in primary screens to represent the structural diversity of mucin side chains, our group discovered several mucin-targeting PUL-like multigenic clusters from a metagenomic library constructed from the mucosal-associated ileal microbiota [26]. These metagenomic PULs, cloned in fosmids and expressed in *E. coli*, encode a cocktail of synergistic CAZymes that were proven, by ELISA assays using fluorescein-conjugated lectins, to affect the structure of human colon mucus sampled from surgical pieces. In order to circumvent the use of clinical samples, validation screening assays and substrate specificity profiling could also be performed using either mucus-secreting human cell lines [41,91], cell-based platforms displaying tunable structures and patterns of *O*-glycans [92], or even – although this has not been performed to date – organoids on a 2D culture system [93,94].

Regarding the rapid identification of mucin-degrading CAZymes, metagenomics has recently been paired with microfluidics, and a fluorogenic glycoside substrate was used in a novel droplet microfluidics workflow developed to directly access metagenomic PULs cloned in fosmids and expressed in *E. coli* [27]. Indeed, droplet microfluidics screening makes it possible to perform 10^6^ assays per hour, with <1 mg of substrates, which is 1000 times faster and less expensive than conventional technologies. This technology is thus compatible with the fast exploration, at low cost, of large sequence spaces to identify the target enzymes. It is based on the detection and sorting of droplet hits in function of their fluorescent or absorbance levels [27,95], bearing in mind that absorbance detection is less sensitive than fluorescence detection by orders of magnitude.

Using such workflows requiring a chemically modified substrate to identify the droplet where the target enzymatic reaction occurs, validation of activity on non-chemically modified substrates (such as commercial oligosaccharides with defined structures, including HMOs and blood antigens, that are both mucin-like motifs) is crucial. In any case, profiling substrate specificity using such structurally defined oligosaccharides, is highly expensive. In primary screening, it is thus very tempting to use such mucin-like oligosaccharides with defined structures, which represent the structural complexity and diversity of physiological substrates. It would prevent screening bias and the costly and time-consuming validation steps on real substrates. Of course, screening large libraries with such oligosaccharides using solid-plates or micro-plate screens is not conceivable, given the price of these substrates. However, recently some ultra-miniaturized droplet-based microfluidics workflows have been developed, based on positive selection of bacteria (recombinant [96] or native bacteria [97]) on native, non-chemically modified oligosaccharides. Until now, these technologies have only been exemplified for the activities of metabolization of plant-derived dietary fibers. What is more, they have not so far led to the discovery of novel functional genes, since the proofs of concepts have been established with a mini-metagenomic library, or with a microbiota sample, for which the taxonomical marker genes have only been sequenced after selecting fiber-metabolizing bacteria. Nevertheless, the development of these workflows opens some very interesting prospects for ultra-high throughput functional genomics, metagenomics and culturomics studies (using single cells or bacterial consortia), targeting the activities of mucin degradation.

## Microbiota-host-diet cross-talk in IBD: the intestinal mucus layer as a key feature

Several approaches have been used over the last decade to study the links between diet, gut bacteria and their enzymes, mucus structure/penetrability, and IBD [98]. In particular, key relationships have been established between the protease activities produced by both intestinal epithelium and opportunistic pathogens, mucosal biofilm structure, and IBD.

**Figure 1:**
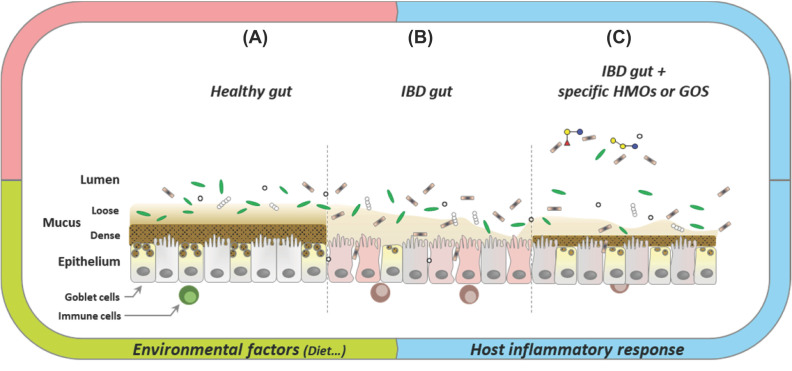
Schematic representation of bowel intestinal epithelium and lumen in healthy (A), IBD condition (B) or IBD condition in presence of HMOs or GOS (C) In the healthy condition (**A**), both mucus layers (in dark, inner and sterile layer and light brown, outer layer) are present. Immune cells are present but not activated. In this condition, mucus secretion and mucus degradation by microbiota are balanced. In the IBD condition (**B**), mucus thickness is highly reduced and both mucus layers are invaded by microbiota, epithelial cell functions are impaired with loss of tight junctions. Immune cells are activated and dysbiosis is observed. Bacteria possibly infiltrate the epithelium. The origin of this unbalanced state could be defects in mucin *O*-glycosylation, resulting in lower density, higher penetrability and degradability of the inner mucus gel, the secretion of which is also affected [12,14]. With a diet supplemented with Human Milk Oligosaccharides (HMOs) and/or Galacto-Oligosaccharides (GMOs) in IBD condition (**C**), an improvement of the situation might be observed. Key: green bars and gray circles: commensal bacteria; dark bars: pathogenic bacteria.

Regarding CAZymes from the intestinal microbiome, pioneer transcriptomics studies targeting *B. thetaiotamicron* (*Bth*) revealed the potential role of these enzymes in gut inflammation, in relationship with diet. Indeed, mucin-targeting *Bth* PULs were shown to be up-regulated in fiber-deprived diets, leading to an alteration of the mucus layer, which promoted the implantation of pathogens and/or commensal opportunists [5,99]. These PULs were also shown to be up-regulated with some particular prebiotics, such as β-galacto-oligosaccharides (GOS), and HMOs [80,100]. GOS are plant-derived oligosaccharides, but as *O*-mucins, they contain β-linked galactosyl units that could be recognized and hydrolyzed by some β-galactosidases or promiscous β-hexosidases or -hexosaminidases of the gut microbiome. GOS have been tested as prebiotics on experimental colitis in rats, but no impact on inflammation was observed [101]. Moreover, the effects of other prebiotics containing β-linked galactosyl units, such as lactulose, on bacterial translocation and inflammation are still unclear [32]. In contrast, consumption of HMOs, which are much more similar to mucins than GOS, seems to protect against IBD [28–30]. Due to their structural convergence, HMOs are indeed thought to act as excellent competitor substrates of mucins towards mucin-degrading CAZymes [102] (Figure 1). In addition, certain HMOs, such as 2′-fucosyllactose, can competitively inhibit the binding of enteric pathogens to epithelial cell membranes, thus modulating specific pro-inflammatory signaling molecules [30]. In healthy adults, 2′-fucosyllactose is well tolerated and promotes the growth of beneficial bifidobacteria [103]. Furthermore, infants fed with a formula containing 2′-fucosyllactose have lower inflammatory cytokine levels. In contrast, other HMOs, such as 3′-sialyllactose, have been shown to directly interact with the host, resulting in structural modification of glycans, or even in pro-inflammatory stimulation [31]. In conclusion, with such a diversity of structures and mechanisms of interaction with both the gut microbiota and the host [104], the effect of functional oligosaccharides like GOS prebiotics and HMOs in the triggering or in the prevention of inflammation is today unclear, and too few CAZymes targeting these motifs in the intestinal microbiome have been characterized to date.

## Summary

- There is a clear correlation between IBD, alteration of the colonic mucus and its barrier effect, and dysbiosis of the intestinal microbiota.
- To date, a few hundred mucus-degrading CAZymes from about ten prevalent gut bacterial species have been biochemically characterized, and only a handful of CAZymes active on mucus or mucin-motifs have been identified from uncultured bacteria, demonstrating the lack of knowledge about mucin catabolic pathways in the gut microbiome.
- The difficulties of mucus sampling, mucus interspecies structural diversity, and the cost and availability of oligosaccharides that properly mimic mucin structure, limit the identification and biochemical characterization of mucin-degrading CAZymes, and of their synergistic action to break down the mucus network.
- Novel droplet microfluidics-based screening technologies developed recently are a great opportunity to explore, at ultra-high throughput and at low cost, the mucin-degrading functions of the gut microbiome, using either functional genomics, metagenomics or culturomics.
- Some genes encoding mucin-degrading CAZymes are more prevalent in the microbiome of IBD patients compared with that of healthy individuals, although the role played by these biomarkers in inflammation has never been studied.
- The action of mucin-targeting CAZymes on mucus invasion by gut bacteria and on the host response should be explored, using a combination of *in vitro*, *ex vivo* and *in vivo* approaches specifically designed to analyze mucus characteristics and inflammation. Also, the protective role of functional foods, more specifically that of prebiotics and HMOs competing with mucin glycans towards mucus-degrading CAZymes, should be assessed. Such holobiomics studies should help demonstrate the potential causality links between mucus degradation, its prevention through functional foods, and the control of the inflammatory response of the host.

## Abbreviations

CAZymes: carbohydrate-active enzymes
Gal: galactose
GalNAc: *N*-acetyl-d-galactosamine
Glc: glucose
GlcNac: *N*-acetyl-d-glucosamine
GH: glycoside hydrolase
GOS: β-galacto-oligosaccharides
HMO: human milk oligosaccharide
IBD: inflammatory bowel diseases
MUC: mucin
Neu5Ac: *N*-acetylneuraminic acid
